# Earthworm species in *Musa* spp. plantations in Brazil and worldwide

**DOI:** 10.3897/zookeys.1033.54331

**Published:** 2021-04-22

**Authors:** Marcus Vinicius Cremonesi, Alessandra Santos, Danilo Eduardo Rozane, Marie Luise Carolina Bartz, George Gardner Brown

**Affiliations:** 1 Universidade Federal do Paraná, Rua dos Funcionários, 1540, Curitiba, PR, 80035-050, Brazil Universidade Federal do Paraná Curitiba Brazil; 2 Universidade Estadual Paulista Júlio de Mesquita Filho, Av. Nelson Brihi Badur, 430, Registro, SP, 11900-000, Brazil Universidade Estadual Paulista Júlio de Mesquita Filho Registro Brazil; 3 University of Coimbra, Calçada Martim de Freitas, Coimbra, 3000-456, Portugal University of Coimbra Coimbra Portugal; 4 Embrapa Forestry, Estrada da Ribeira, Km. 111, Colombo, PR, 83411-000, Brazil Embrapa Forestry Colombo Brazil

**Keywords:** Annelida, banana, biodiversity, Oligochaeta, plantain, *Pontoscolex corethrurus*

## Abstract

Bananas and plantains are major commodity/food crops that represent an important habitat for earthworms, although so far, no review is available on earthworm communities associated with banana/plantain crops worldwide. The Vale do Ribeira region is among the largest banana producing areas in Brazil, but little is known of the earthworms living there. Hence, the present study assessed earthworm populations and species in three banana plantations and adjacent Atlantic forest fragments along the Ribeira de Iguape River using standard (hand sorting) methodologies. Furthermore, we review earthworm populations reported in banana/plantain plantations worldwide. Only two species (*Pontoscolex corethrurus*, *Amynthas gracilis*) belonging to two families (Rhinodrilidae, Megascolecidae) were found in the Ribeira River valley, occurring concurrently. Abundance was low (< 13 indiv. m^-2^) compared with other banana plantations worldwide, that frequently surpassed 100 indiv. m^-2^. More than 70 studies reported earthworms from >200 banana plantations in 28 countries, and mean species richness was 2.7 per site, ranging from 1 to 10 species. Exotics predominated in most sites and *P. corethrurus* was the most prevalent species encountered. Overall, more than 104 species from 10 families were reported, with around 61 native and 43 exotic widespread species, mainly of the Megascolecidae, Lumbricidae and Acanthodrilidae families. Richness was highest in India (27 spp.) and the Canary Islands (25 spp.), but native species dominated only in a few countries and sites, while exotics were prevalent especially in island countries and Brazil. Lower-input practices appear to be important for earthworm communities and banana plantations can have large earthworm populations in some cases, which may be contributing to soil processes and plant production, topics that deserve further attention. However, many important banana-producing countries have not yet been evaluated, so further work is warranted, both in terms of applied ecology and biodiversity.

## Introduction

Bananas and plantains are large, perennial herbs belonging to the genus *Musa*, that evolved in Indochina and Southeast Asia, but with major secondary diversification in Africa, India and the Caribbean (Price 1995). Bananas are a major commodity, occupying over 6 million ha (FAO 2018) and representing an important contribution to the economy of many developing countries worldwide (OECD/FAO 2019). Plantains resemble bananas, but are generally longer, have more starch and are mostly eaten cooked, rather than raw (like the bananas). They are a major staple crop in several African, Asian, Pacific, Latin American and Caribbean countries (Price 1995; Norgrove and Hauser 2014). In 2018, the six main banana producers (total production) were India, China, Indonesia, Brazil, Ecuador and the Philippines, while the six countries with the greatest surface area devoted to banana production were India (884,000 ha), Tanzania (490,701 ha), Philippines (484,247 ha), Rwanda (464,321 ha), Brazil (449,284 ha) and China (383,216 ha) (FAO 2018). India accounts for around 24% of global production and Brazil around 5% (FAO 2018), while the whole of Latin America and the Caribbean (LAC) region account for around 25% of the world’s banana production (OECD/FAO 2019).

Throughout much of LAC, bananas and plantains are still cultivated at the subsistence level, often in agroforestry systems (Harvey and Villalobos 2007; Malézieux et al. 2009; Paul et al. 2015; Coelho 2017; Garcia et al. 2017; Salazar-Díaz and Tixier 2017). However, commercial plantations are also widespread, occupying large monoculture areas, particularly in warmer, wetter regions of the tropics (Campbell 2018; Yahia 2019). In Brazil, most of the area devoted to banana cultivation lies within the Atlantic Rainforest biome, a highly threatened hotspot of biodiversity (Myers et al. 2000). In fact, much of the banana and plantain cultivation worldwide is performed in wetter tropical climates, and frequently close to rainforest ecosystems, where they may represent a potential hazard to biodiversity conservation. In commercial plantations, conventional production practices are adopted, including frequent herbicide use to control weeds, fumigation to control fungal diseases (particularly *Fusarium* and *Pythium*) and root nematode infestation, as well as Sigatoka (Marin et al. 2003; Cordeiro et al. 2004; Gasparotto et al. 2006), although some resistant varieties for the latter are already available (Timm et al. 2016; Dale et al. 2017). These practices may have important negative impacts on earthworm populations (da Silva et al. 2006; Baretta et al. 2011), despite the high amounts of litter inputs, which represent C (food) sources for soil biota, and protection from soil erosion (Lombardi Neto and Moldenhauer 1992). Worldwide, however, little is known of the soil biota inhabiting banana plantations, and so far, there has not been an overview of true soil-inhabiting animals in banana plantations worldwide.

Earthworms are essential service providers for terrestrial ecosystems (Lavelle et al. 2006). Their activity, generating galleries and casts, contributes to formation and maintenance of soil structure (Lavelle 1997; Capowiez et al. 2012), increasing porosity, infiltration and water retention (Fiuza et al. 2012), as well as re-distribution and breakdown of soil organic matter (Brown et al. 2000). However, earthworms are sensitive to land use and management, and can be used as soil quality and management as well as environmental bioindicators (Brown and Domínguez 2010; Bartz et al. 2013; Bünemann et al. 2018). Brazil is home to more than 300 described earthworm species (Brown et al. 2013), but practically nothing is known of the species and populations inhabiting banana plantations in the country.

The Vale do Ribeira region, located in northeastern Paraná State and southern São Paulo State, has extensive areas (over 36,000 hectares; ABAVAR 2015) devoted to banana cultivation (Bueno 2003). In this region, banana fields are normally surrounded by Atlantic forest fragments (Cordeiro et al. 2017), that have been reduced to around 12% of their original surface area (Ribeiro et al. 2009). Although frequently disturbed with various management practices, banana plantations are perennial crops that could provide adequate habitats for the establishment of native earthworm species, especially when Atlantic forest fragments occur surrounding banana cropping areas (Cordeiro et al. 2017). However, little is known about the effects of banana crops on abundance and diversity of earthworm species, and the occurrence of these invertebrates in Atlantic forest fragments in the Ribeira valley region. Furthermore, little is known of the presence of native and exotic earthworm species in banana and plantain fields worldwide. Hence, the present study was undertaken to assess earthworm populations in banana plantations and native forest fragments in the Ribeira de Iguape River valley in the State of São Paulo, and evaluate earthworm communities (abundance, biomass, species composition) associated with banana and plantain crops worldwide.

## Material and methods

### Study sites in the Ribeira de Iguape River valley

**Figure 1.**
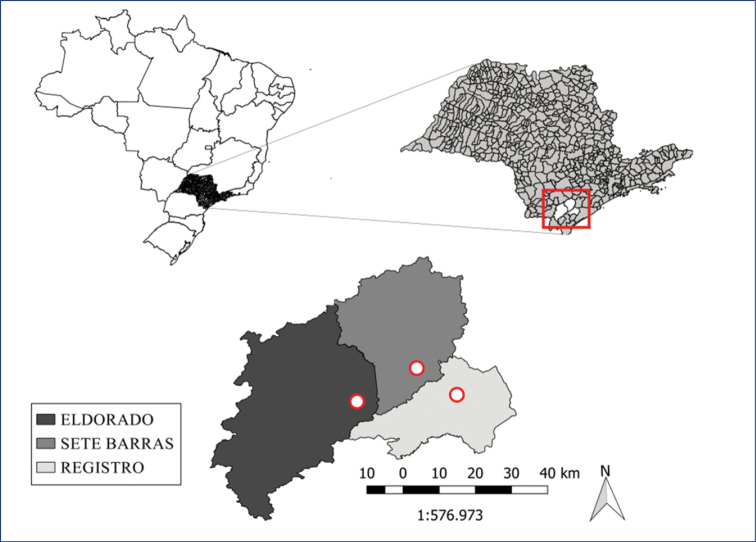
Location of the counties sampled in the Ribeira de Iguape River valley, São Paulo State, Brazil.

Three counties in the lower Ribeira River valley, all of them in the State of São Paulo were selected for this study: Eldorado, Sete Barras and Registro (Fig. 1). The climate in Sete Barras and Registro is rainy tropical (Af-type according to Köppen), with mean rainfall greater than 60 mm in the driest month. In Eldorado, climate is Köppen Am tropical, with rainfall less than 60 mm in the driest month. The average annual rainfall for all counties ranges from 1500 to 1600 mm (CEPAGRI 2018; CIIAGRO 2018), with the highest concentration of rains occurring from January to March. The mean annual temperature ranges from 23.9 to 24.3 °C, with the lowest temperature (13 °C) in July and highest (34.2 °C) in February. Soils in the valley originate from sedimentary, metabasic and amphibolic rocks (Oliveira et al. 2002), with high natural fertility (calcium, magnesium, potassium, and phosphorus content) and high organic matter levels, due to seasonal river floods that deposit alluvial material. Soil texture varies from loam to clay. The areas chosen in the three counties are characterized by smaller watersheds that flow into the Ribeira River with banana crops on the high ground level and Atlantic forest sites (control sites) in advanced stages of regeneration close to the Ribeira River. General characteristics of the areas are given in Table 1.

**Table 1.** Land use system, watershed number (WN), age of the land use, geographic coordinates and soil types according to FAO classification (IUSS/WRB 2015) of the sites evaluated in each county of the Ribeira de Iguape River Valley, São Paulo, Brazil.

Site	County	System	WN^1^	Age (yrs)	Latitude, Longitude	Soil types
1	Eldorado	Banana	344	50	24°29'35"S, 48°02'10"W	Cambisols
2	Eldorado	Atlantic forest	344	> 50	24°30'09"S, 48°02'30"W	Cambisols
3	Sete Barras	Banana	422	15	24°23'34"S, 47°53'51"W	Cambisols
4	Sete Barras	Atlantic forest	422	> 50	24°23'30"S, 47°53'22"W	Cambisols
5	Registro	Banana	379	40	24°26'56"S, 47°49'41"W	Cambisols / Histosols
6	Registro	Atlantic forest	389	45	24°26'47"S, 47°49'23"W	Cambisols / Histosols

^1^Official cartographic number for the watershed.

### Earthworm sampling

Earthworms were collected using an adaptation of the standard sampling method proposed by the Tropical Soil Biology and Fertility (TSBF) Programme (Anderson and Ingram 1993). In each area 10 samples (25 × 25 cm square to 20 cm depth) were taken, divided into 2 equally-numbered transects with samples every 20 m. Distance between transects was ca 10 m. Earthworms were hand-sorted from the soil in the field and fixed in 80% alcohol. In the laboratory, earthworms were identified to species or family level (juveniles) using taxonomic keys (Michaelsen 1900; Righi 1990; Blakemore 2002). The material was deposited in the Fritz Müller Oligochaete collection (COFM) at Embrapa Forestry in Colombo, Brazil. The earthworm data obtained were used to determine the total species abundances (no. individuals and fresh mass m^-2^) and richness, per site and land use (banana, forest).

### Literature review

Both the common and scientific names of banana were used for a bibliographic search online using the keywords for bananas and plantains in English, Portuguese, French and Spanish: *Musa* (genus), *Musa acuminata*, *Musa balbisiana*, banana, banane, banano, plátano and plantain. These were then crossed with the common names of earthworms in these languages: earthworms, minhoca, oligochaeta, oligoqueta, vers de terre and lombriz de tierra. Online scientific databases Web of Science, Science Direct, Scielo, google academic and the Base de Dados de Teses e Dissertações (BDTD – Thesis and Dissertation Database) of Brazil were consulted. All the resulting publications were consulted and those containing data on earthworm abundance (density and/or biomass) or species identification were selected and these data extracted, as well as information on sampling sites (counties, countries, management practices of the plantations). Earthworm species were separated into different families and into native or exotic to the region of occurrence, and species richness per site and for each group (native, exotic), when available. Although we treated bananas and plantains separately when possible, for most of the analysis we considered them together, since not all publications provided details regarding the types of bananas cultivated, and even plantains are often called ‘bananas.’ Details on the species and management data obtained and presented in this paper are available for download online from the open access repository Mendeley Data at http://dx.doi.org/10.17632/p8ywsnj8c5.1 (Cremonesi et al. 2020).

## Data treatment

Quantitative data on the earthworm abundance and biomass obtained from the literature and from the present study were treated as follows. Means of earthworm abundance (no. individuals m^-2^) and biomass (fresh mass in gm^-2^) were calculated per sampling site (plantation), using data from the present study. When quantitative data from the literature was available for the individual site, it was used as is. When only means for several plantations in the same general location were provided, these were also used. As the interest of the present study was more at the spatial (site-level) rather than the temporal scale, when samples were taken on multiple occasions, and individual means per sampling date were not available, overall means were used. When taken in wet and dry seasons, both values were used as an interval of abundance and biomass (when measured).

## Results and discussion

### Specimens examined from the Ribeira de Iguape River valley sites

#### Family Rhinodrilidae

**Pontoscolex (Pontoscolex) corethrurus (Müller, 1857)**

COFMBRSP0231, 1 individual in Atlantic Forest, HMN 389, Registro – SP (24°26'16.85"S, 47°49'31.71"W), 2019, M. Cremonesi, A. Santos colls. COFMBRSP0232, 2 individuals in Atlantic Forest, HMN 389, Registro – SP (24°26'16.82"S, 47°49'31.71"W), 2019, M. Cremonesi, A. Santos colls. COFMBRSP0233, 2 individuals in Atlantic Forest, HMN 389, Registro – SP (24°26'16.28"S, 47°49'32.52"W), 2019, M. Cremonesi, A. Santos colls. COFMBRSP0235, 2 individuals in Atlantic Forest, HMN 389, Registro – SP (24°26'15.71"S, 47°49'33.32"W), 2019, M. Cremonesi, A. Santos colls. COFMBRSP0236, 1 individual in Atlantic Forest, HMN 389, Registro – SP (24°26'14.57"S, 47°49'35.35"W), 2019, M. Cremonesi, A. Santos colls. COFMBRSP0238, 2 individuals in banana field, HMN 379, Registro – SP (24°26'54.25"S, 47°49'38.12"W), 2019, M. Cremonesi, A. Santos colls. COFMBRSP0239, 1 individual in banana field, HMN 379, Registro – SP (24°26'54.81"S, 47°49'39.41"W), 2019, M. Cremonesi, A. Santos colls. COFMBRSP0240, 1 individual in Atlantic Forest, HMN 422, Sete Barras – SP (24°23'44.43"S, 47°55'11.56"W), 2019, M. Cremonesi, A. Santos colls. COFMBRSP0241, 1 individual in Atlantic Forest, HMN 422, Sete Barras – SP (24°23'44.46"S, 47°55'11.49"W), 2019, M. Cremonesi, A. Santos colls. COFMBRSP0242, 2 individuals in Atlantic Forest, HMN 422, Sete Barras – SP (24°23'43.79"S, 47°55'24.53"W), 2019, M. Cremonesi, A. Santos colls. COFMBRSP0244, 1 individual in Atlantic Forest, HMN 422, Sete Barras – SP (24°23'43.93"S, 47°55'10.17"W), 2019, M. Cremonesi, A. Santos colls. COFMBRSP0245, 3 individuals in Atlantic Forest, HMN 422, Sete Barras – SP (24°23'44.33"S, 47°55'09.65"W), 2019, M. Cremonesi, A. Santos colls. COFMBRSP0248, 1 individual in Atlantic Forest, HMN 422, Sete Barras – SP (24°23'44.90"S, 47°55'08.92"W), 2019, M. Cremonesi, A. Santos colls. COFMBRSP0249, 1 individual in banana field, HMN 422, Sete Barras – SP (24°23'38.61"S, 47°55'23.49"W), 2019, M. Cremonesi, A. Santos colls. COFMBRSP0251, 1 individual in banana field, HMN 422, Sete Barras – SP (24°23'43.01"S, 47°55'24.52"W), 2019, M. Cremonesi, A. Santos colls. COFMBRSP0252, 3 individuals in banana field, HMN 422, Sete Barras – SP (24°23'42.54"S, 47°55'25.32"W), 2019, M. Cremonesi, A. Santos colls. COFMBRSP0253, 1 individual in Atlantic Forest, HMN 344, Eldorado – SP (24°29'57.34"S, 48°02'41.68"W), 2019, M. Cremonesi, A. Santos colls. COFMBRSP0255, 1 individual in Atlantic Forest, HMN 344, Eldorado – SP (24°29'55.69"S, 48°02'42.15"W), 2019, M. Cremonesi, A. Santos colls. COFMBRSP0256, 2 individuals in banana field, HMN 344, Eldorado – SP (24°29'36.89"S, 48°02'09.43"W), 2019, M. Cremonesi, A. Santos colls. COFMBRSP0258, 2 individuals in banana field, HMN 344, Eldorado – SP (24°29'37.11"S, 48°02'10.84"W), 2019, M. Cremonesi, A. Santos colls.

**Rhinodrilidae juveniles.** COFMBRSP0246, 1 individual in Atlantic Forest, HMN 422, Sete Barras – SP (24°23'44.33"S, 47°55'09.65"W), 2019, M. Cremonesi, A. Santos colls.

#### Family Megascolecidae

***Amynthas gracilis* (Kinberg, 1867)**

COFMBRSP0237, 1 individual in banana field, HMN 379, Registro – SP (24°26'54.25"S, 47°49'38.22"W), 2019, M. Cremonesi, A. Santos colls. COFMBRSP0250, 3 individuals in banana field, HMN 422, Sete Barras – SP (24°23'38.61"S, 47°55'23.49"W), 2019, M. Cremonesi, A. Santos colls.

**Megascolecidae juveniles.** COFMBRSP0234, 1 individual in Atlantic Forest, HMN 389, Registro – SP (24°26'16.28"S, 47°49'32.52"W), 2019, M. Cremonesi, A. Santos colls. COFMBRSP0243, 1 individual in Atlantic Forest, HMN 422, Sete Barras – SP (24°23'44.06"S, 47°55'10.35"W), 2019, M. Cremonesi, A. Santos colls. COFMBRSP0247, 1 individual in Atlantic Forest, HMN 422, Sete Barras – SP (24°23'44.33"S, 47°55'09.65"W), 2019, M. Cremonesi, A. Santos colls. COFMBRSP0254, 1 individual in Atlantic Forest, HMN 344, Eldorado – SP (24°29'56.60"S, 48°02'42.23"W), 2019, M. Cremonesi, A. Santos colls. COFMBRSP0257, 1 individual in banana field, HMN 344, Eldorado – SP (24°29'36.89"S, 48°02'09.43"W), 2019, M. Cremonesi, A. Santos colls.

### Earthworm populations in the Ribeira River valley and other sites in Brazil

Only two earthworm species belonging to two families (Rhinodrilidae, Megascolecidae) were found at the six sampling sites in the three counties (Table 2): Pontoscolex (Pontoscolex) corethrurus and *Amynthas gracilis*, both considered peregrine/exotic in southern Brazil (Brown et al. 2006). *Pontoscolex corethrurus* may have originated in the Guyana shield area (Righi 1984), and *A. gracilis* may be native to China (Blakemore 2002). The former species was found living in all sites, while the latter was found in both banana plantations and native forest in Sete Barras and in banana plantations in Registro. At the other sites, only juveniles of the Megascolecidae family were found. These were most likely *A. gracilis* as well, but could not be identified to species level. Maximum richness found per site was similar in banana crops and Atlantic forest fragments (two spp. in each land use), but with some variation between sites (Table 2).

**Table 2.** Earthworm families, species, and richness in banana plantations and Atlantic Forest remnants, in three counties of the Ribeira de Iguape River valley (Eldorado, Sete Barras, Registro). + means presence and – means absence.

Earthworm family and species	Eldorado		Sete Barras		Registro	
	Banana	Atlantic Forest	Banana	Atlantic Forest	Banana	Atlantic Forest
** Megascolecidae **						
*Amynthas gracilis*	–	–	+	+	+	–
Megascolecidae juveniles	+	+	–	+	–	+
** Rhinodrilidae **						
*Pontoscolex corethrurus*	+	+	+	+	+	+
Rhinodrilidae juveniles	–	–	–	+	–	–
**Species Richness**	2	2	2	≥2	2	2

**Figure 2.**
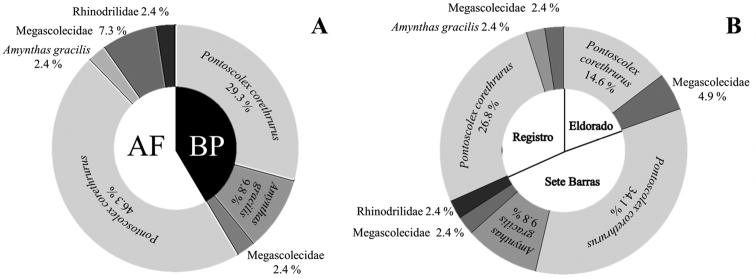
Frequency of earthworm species (% of total individuals collected) in each ecosystem sampled (**A**) in Atlantic Forest (AF) and banana plantations (BP) and by counties (**B**).

Most of the individuals collected (76% of the total) were of *P. corethrurus*, representing 29% of the total abundance in banana crop sites and 46% in Atlantic forest fragments (Fig. 2). *Amynthas gracilis*, although not occurring in all areas, accounted for 12% of all individuals sampled, of which 10% were found in banana crops but only 2% in Atlantic forests. Rhinodrilidae juveniles represented only 2% of the earthworms found, and occurred only in the Atlantic forest, while Megascolecidae juveniles represented 10% of all earthworms, and were often found in Atlantic forest fragments. Both species are widespread in Brazil (Brown et al. 2006), especially in agricultural and disturbed ecosystems, and display relatively high tolerance to a range of abiotic/biotic conditions, which have allowed these species to spread throughout most of the tropics and subtropics worldwide (Brown et al. 2006; González et al. 2006; Taheri et al. 2018). They have also been recommended as indicators of soil quality in agroecosystems and of disturbance in natural landscapes (Nunes et al. 2007; Fernandes et al. 2010).

The predominance of *P. corethrurus* in both native forest and banana plantations of the Ribeira River valley indicate that non-native species have extensively colonized disturbed soils of this region. Nonetheless, this potentially widespread occurrence of exotics should be further evaluated both regionally and nationally, in order to better determine the extent of this phenomenon as well as its possible causes.

Mean overall abundance and biomass of earthworms found in the three sites studied here (6 to 13 indiv. m^-2^ and 2.5 to 9 g m^-2^) tended to be quite low compared with others observed overall in Brazil (21 to 459 indiv. m^-2^ and 3.1 to 177.4 g m^-2^; see Table 3). At sites near the Ribeira River valley in the neighboring state of Paraná (Römbke et al. 2009; Maschio et al. 2010), and within the Ribeira River watershed in the nearby Turvo River valley (a tributary of the Ribeira River; Brown et al. 2009), both abundance and biomass were generally much higher (Table 3), even though the predominant earthworm species was the same (*P. corethrurus*). This is probably due to the less intensive and more traditional agroforestry management practices used in these sites, including slashing and mulching, as well as the presence of other trees, particularly atmospheric N_2_-fixing leguminous trees, and the absence of or lower pesticide use (Brown et al. 2009; Römbke et al. 2009). These practices may benefit earthworm populations, particularly *P. corethrurus*, as observed comparing a mulched and non-mulched plantation in Antonina, where earthworm abundance was ~13 times higher with mulching (Maschio et al. 2010). Reasons for the lower values found in the Ribeira River valley sites may be due to the more intensive management practices typical of commercial banana plantations in the region, including insecticide and nematicide applications, which may reduce earthworm populations (Clermont-Dauphin et al. 2004).

**Table 3.** Earthworm abundance and biomass found in banana plantations worldwide, and the predominant species encountered (when available).

Country	Location	Abundance (indiv. m^–2^)	Biomass (g m^–2^)	Predominant species	References
Brazil	Antonina (Monoculture)	71	35.1	*P. corethrurus*	Römbke et al. (2009)
		221	95.7	*P. corethrurus*	
		86	23.8	*P. corethrurus*	
	Antonina (Agroforestry)	173	77.1	*P. corethrurus*	
		338	69.6	*P. corethrurus*	
		117	43.5	*P. corethrurus*	
		21^a^	3.1^b^	*P. corethrurus*	Maschio et al. (2010)
		293^a^	34.9^b^	*P. corethrurus*	
	Adrianópolis (Agroforestry)	211–413^c^	37–71.2^c^	*P. corethrurus*	Brown et al. (2009)
	Barra do Turvo (Agroforestry)	99–176^c^	11.2–17.3^c^	*P. corethrurus*	
		229–459^c^	48.3–117.4^c^	*P. corethrurus*	
	Casimiro de Abreu	~205–440^c^	–	NA	Quintero (2010)
	Paraty	167	–	NA	Correia et al. (2001)
	Eldorado	8	3.9	*P. corethrurus*	This study
	Sete Barras	13	9.0	*P. corethrurus*	
	Registro	6	2.5	*P. corethrurus*	
Cameroon	Mbalmayo Forest Reserve	70	–	*Legonodrilus* sp. nov. 1, *Eminoscolex lamani*	Norgrove et al. (2011)
		121	–	*Legonodrilus* sp. nov. 1, *Eminoscolex lamani*	
	Campo Ma’an	16–92^d^	–	NA	Kanmegne (2004)
Colombia	Quindío (Armenia)	9–16^e^	1.2–3.0^e^	NA^f^	Molina and Feijoo (2016)
Costa Rica	Limón Province (Finca San Pablo)	83–812^g^	–	NA	Agüero et al. (2002)
	Pueblo Nuevo de Villa Franca de Guácimo, Limón	29	6.2	NA	Cornwell (2014)
	Cahuita	350	144.6	*P. corethrurus*	Lapied and Lavelle (2003)
Guadeloupe (France)	Basse-Terre Andosols (mean of 23 sites)	88	23	NA	Clermont-Dauphin et al. (2004)
	Basse Terre Nitisols (mean of 11 sites)	54	17.5	NA	
	Capesterre-Belle-Eau (Gloria Bas)	168	27.6	*P. corethrurus*	Burac et al. (2018)
	Capesterre-Belle-Eau (Source)	288	42.2	*P. corethrurus*	
	Capesterre-Belle-Eau (Bergerie)	188	33.6	*P. corethrurus*	
	Baillif (Sextius)	336	112	*P. corethrurus*	
	Baillif (Grand Canon)	192	70.8	*P. corethrurus*	
	Saint-Claude (Saut d’Eau)	364	46	*P. corethrurus*	
Ecuador	Latacunga (La Maná)	168	–	NA	Avilés (2017)
		111	–	NA	
	Manabí (El Carmen)	78	–	NA	
		37	–	NA	
	El Carmen (Cijádi)	0–145^h^	–	NA	Figueroa (2019)
	El Carmen (Nápoles)	34–144^h^	–	NA	
	Santo Domingo de los Tsáchilas (Santa Patrícia)	83–548^h^	–	NA	
	Santo Domingo de los Tsáchilas (La Floresta)	22–150^h^	–	NA	
India	West Tripura	16–656^i^	4.8–453.6^i^	*P. corethrurus*	Dhar and Chaudhuri (2018)
	Rajapalayam	116	48.8	*Lampito mauritii*, *Perionyx excavatus*	Mariappan et al. (2013)
Ivory Coast	Taabo (Lamto reservation)	186	8.5	*Reginaldia anomala*	Tondoh (1994, 2007)
Martinique (France)	Le Lorrain (Feugère)	244	67.6	*P. corethrurus*	Burac et al. (2018)
	Le Lorrain (Bellevue)	152	43.6	*P. corethrurus*	
	Le Lorrain (Limite)	52	26	*P. corethrurus*	
	L’Ajoupa-Bouillon (Allée Domergue 3)	148	49.6	*P. corethrurus*	
	Basse-Pointe (Fromager Rivière)	80	26	*P. corethrurus*	
	Basse-Pointe (Dantu Bas)	40	9	*P. corethrurus*	
Mexico	Tabasco, Pablo L. Sidar	25	10	*P. corethrurus*, *Lavellodrilus bonampakensis*	Huerta et al. (2005)
	Tabasco, Teapa	116	20.8	*P. corethrurus*, *Drawida barwelli*, *Polypheretima elongata*	Geissen et al. (2009)
		117	11.8	*Balanteodrilus pearsei*, *Drawida barwelli*	
		94	40.4	*Balanteodrilus pearsei*, *Polypheretima elongata*	
		125	35.6	*P. corethrurus*, *Drawida barwelli*	
		25	8.8	*P. corethrurus*, *Lavellodrilus bonampakensis*	Huerta et al. (2007)
		~350	2.5	*Diplotrema murchiei*	Huerta et al. (2013)
		~350	9.3	*P. corethrurus*	
		~470	16.2	*P. corethrurus*	
		~100	11	*P. corethrurus*	
		~80	2.8	*P. corethrurus*	
		~125	0.8	*Dichogaster* sp.	
Nicaragua	León (Finca Cony)	150	–	NA	Hernández et al. (2015)
	León (Finca San Martín)	325	–	NA	
	León (Finca Santa Isabel)	50	–	NA	
	León (Finca El verdon)	65	–	NA	
	Possoltega (Finca San Joaquin)	150	–	NA	
	Possoltega (Finca Los Ángeles)	225	–	NA	
	Possoltega (Finca Maria de los Ángeles)	100	–	NA	
	Possoltega (Finca Montes Verdes)	125	–	NA	
Philippines	Davao (Sumitomo Fruits Corporation)	~85–175^j^	–	NA	Fusilero et al. (2013)
		~75–215^j^		*Metaphire cai*	
South Africa	Kwazulu-Natal (Eshowe)	1500^k^	180	*Amynthas rodericensis*, *Amynthas minimus*, *P. corethrurus*	Dlamini and Haynes (2004)
Uganda	Kabanyolo University Farm	18–207^l^	0.1–9.4^l^	*Dichogaster* sp. 2, *Gordiodrilus* sp. 1	Block and Banage (1968)
	Mabira Forest reserve (1 yr old)	13	0.4	NA	Okwakol (1994)
	(2 yr old)	125	2.2	NA	
	(3 yr old)	131	1.3	NA	
	(5 yr old)	54	0.5	NA	
	(20 yr old)	154	4.2	NA	

^a^Earthworm abundance values were corrected from Maschio et al. (2010) that reported earthworm numbers per sample and not per m^2^. ^b^Biomass values in g m^-2^ are now included for this study. ^c^Mean of dry and wet season samplings, respectively. ^d^Mean abundance from eight sites, with four sampled in one year and the other four the subsequent year. ^e^Range of abundance taken from eight replicate farms under four different management practices (totaling 32 plantations) in the Armenia region. ^f^The identification of the earthworm species collected overall in this study (not by plantation type) is published in Feijoo et al. (2018). ^g^Range of abundance found under six weed control treatments (performed on same banana plantation) on five sampling dates; ^h^Range of abundance found on six sampling dates in same plantation. ^i^Range of abundance and biomass found in three banana plantations. ^j^Range of abundance and biomass found on sixteen sampling dates in same plantation. ^k^Mean of six banana plantations. ^l^Range of abundance and biomass found on eight sampling dates in same plantation.

### Earthworm communities in banana plantations worldwide

More than 70 studies were found from 28 countries with data on earthworms in banana and plantain fields (Tables 3, 4, 5). Of these studies, 49 had species data (Table 4; see also full dataset in Cremonesi et al. 2020), coming from ≥ 210 sites (Table 5), of which most were in the Spanish Canary Islands (*N* = 77), mainly due to the intensive sampling efforts of Talavera in Tenerife (Talavera 1992a). Interestingly, two of the major banana-producing countries in terms of area were not represented (Tanzania, Rwanda), and in China (another important producer), only one study reported earthworms from a single site (Sun et al. 2012). Plantain banana fields were sampled in only 22 locations (10% of total) in four countries (Colombia, Cameroon, Ivory Coast and Ecuador; Tondoh 2007; Norgrove et al. 2011; Avilés 2017; Feijoo et al. 2018), and involved traditional management practices, rather than conventional cultivation. Most of the fields evaluated were banana plantations, and only in Ecuador were mixed banana/plantain fields evaluated (Avilés 2017).

**Table 4.** Earthworm species, richness and number of native and exotic species found in banana plantations under various management practices worldwide.

Country	Location	Management	Culture type	Earthworm species	Richness	Native (N) /Exotic (E)	References
**Bangladesh**	Lalmonirhat District	NA	NA	*Lampito mauritii*, *Metaphire posthuma*, *Pontoscolex corethrurus*	3	2/1	Reynolds et al. (1995)
**Bermuda**	Paget Parish	NA	NA	*Amynthas rodericensis*	1	0/1	Reynolds and Fragoso (2004)
	Southampton Parish	NA	NA	*Amynthas hupeiensis*	1	0/1	Reynolds and Fragoso (2004)
**Brazil**	Antonina, PR	Agroforestry	Monoculture	*Amynthas corticis*, *Pontoscolex corethrurus*, two other spp.	4	0/4	Römbke et al. (2009)
	Antonina, PR	Agroforestry	Monoculture	*Amynthas gracilis*, *Pontoscolex corethrurus*, two other spp.	4	0/4	Römbke et al. (2009)
	Antonina, PR	Agroforestry	Monoculture	*Dichogaster* spp., *Pontoscolex corethrurus*	4	0/4	Römbke et al. (2009)
	Antonina, PR	Agroforestry	Monoculture	*Ocnerodrilus occidentalis*, *Pontoscolex corethrurus*, two other spp.	4	0/4	Römbke et al. (2009)
	Antonina, PR	Agroforestry	Monoculture	*Pontoscolex corethrurus*, one other sp.	2	0/2	Römbke et al. (2009)
	Antonina, PR	NA	Polyculture	*Pontoscolex corethrurus*, one other sp.	2	0/2	Römbke et al. (2009)
	Antonina, PR	Agroforestry	Polyculture	*Dichogaster* sp., *Pontoscolex corethrurus*, and one unidentified sp.	3	?/2	Maschio et al. (2010)
	Antonina, PR	Agroforestry	Polyculture	*Pontoscolex corethrurus*	1	0/1	Maschio et al. (2010)
	Adrianópolis, PR	Agroforestry	Polyculture	*Amynthas gracilis*, *Pontoscolex corethrurus*	2	0/2	Brown et al. (2009)
	Barra do Turvo, SP	Agroforestry	Polyculture	*Amynthas gracilis*, *Pontoscolex corethrurus*	2	0/2	Brown et al. (2009)
	Barra do Turvo, SP	Agroforestry	Polyculture	*Amynthas gracilis*, *Dichogaster* sp., *Pontoscolex corethrurus*	3	0/3	Brown et al. (2009)
	Areia, PB	NA	Polyculture	*Amynthas gracilis*, *Dichogaster affinis*, *Eudrilus eugeniae*, *Pontoscolex corethrurus*	4	0/4	Guerra and Silva (1994)
	Eldorado, SP	Conventional	Monoculture	*Amynthas gracilis*, *Pontoscolex corethrurus*	2	0/2	This study
	Jutaí River margin, AM	NA	NA	*Pontoscolex corethrurus*	ND	0/1	Righi (1990)
	Registro, SP	Conventional	Monoculture	*Amynthas gracilis*, *Pontoscolex corethrurus*	2	0/2	This study
	Sete Barras, SP	Conventional	Monoculture	*Amynthas gracilis*, *Pontoscolex corethrurus*	2	0/2	This study
**Cameroon**	Mbalmayo Forest Reserve (low density cover)	Organic Agroforestry	Monoculture	*Dichogaster hauseri*, *Eminoscolex lamani*, *Eudrilidae* gen. et sp. nov.1 & 2, *Legonodrilus* sp. nov.1, *Malodrilus kamerunensis*, *Nematogenia panamaensis*, *Rosadrilus camerunensis*	8	7/1	Norgrove et al. (2011)
	Mbalmayo Forest Reserve (high density cover)	Organic Agroforestry	Monoculture	*Dichogaster annae*, *Dichogaster bolaui*, *Dichogaster* sp., *Eminoscolex lamani*, *Eudrilidae* sp., *Eudrilidae* gen. et sp. nov. 1, *Legonodrilus* sp. nov. 1, *Nematogenia panamaensis*, *Ocnerodrilidae* gen. et sp. nov., *Rosadrilus camerunensis*, *Scolecillus tantillus*	10	7/3	Norgrove et al. (2011)
**China**	Hainan Province	NA	NA	*Pheretima montana*	ND	0/1	Sun et al. (2012)
**Colombia**	Quindío, Circasia, Barcelona (La Sofe farm)	NA	Monoculture	*Aptodrilus fuhrmanni*, *Amynthas minimus*, *Glossodrilus chaguala*, *Glossodrilus panikita*, *Martiodrilus quimbayaensis*	5	4/1	Feijoo et al. (2018)
	Quindío, Circasia, Barcelona (La Sofe farm)	NA	Polyculture	*Aptodrilus fuhrmanni*, *Amynthas minimus*, *Glossodrilus chaguala*, *Glossodrilus panikita*, *Martiodrilus quimbayaensis*	5	4/1	Feijoo et al. (2018)
	Quindío, Circasia, Barcelona (La Sofe farm)	NA	NA	*Amynthas gracilis*, *Periscolex columbianus*	2	1/1	Feijoo et al. (2018)
**Colombia**	Armenia, Niagara (La Catalina)	NA	NA	*Amynthas gracilis*, *Glossodrilus griseus*, *Pontoscolex corethrurus*	3	1/2	Feijoo et al. (2018)
	Quindio, Calarcá, Quebrada Negra	NA	NA	*Glossodrilus griseus*	1	1/0	Feijoo et al. (2018)
	Quindío, Marmato (La Cristalina farm)	NA	Monoculture	*Glossodrilus lacteus*	1	1/0	Feijoo et al. (2018)
	Quindío, Marmato (La Cristalina farm)	NA	Polyculture	*Glossodrilus lacteus*	1	1/0	Feijoo et al. (2018)
	Quindío, Marmato (La Cristalina farm)	NA	NA	*Dichogaster affinis*	1	0/1	Feijoo et al. (2018)
	Armenia, La Revancha (Villa Sofia farm)	NA	NA	*Amynthas gracilis*, *Dichogaster affinis*, *Dichogaster bolaui*, *Glossodrilus griseus*, *Perionyx excavatus*	5	1/4	Feijoo et al. (2018)
	Armenia, La Revancha (Bella Marina farm)	NA	NA	*Dichogaster saliens*, *Periscolex columbianus*	2	1/1	Feijoo et al. (2018)
	Quindío, Armenia, El Rhin	NA	NA	*Periscolex columbianus*	1	1/0	Feijoo et al. (2018)
	Quindío, Armenia, La India (La Ermita farm)	NA	NA	*Periscolex coreguaje*	1	1/0	Feijoo et al. (2018)
	Circasia, Barcelona Baja rural (Buenos Aires farm)	NA	NA	*Amynthas gracilis*, *Dichogaster saliens*, *Pontoscolex corethrurus*	3	0/3	Feijoo et al. (2018)
	Quindío, Armenia, La India (La Miranda farm)	NA	NA	*Dichogaster saliens*	1	0/1	Feijoo et al. (2018)
	Quindío, Armenia, La Patria	NA	NA	*Dichogaster saliens*	1	0/1	Feijoo et al. (2018)
**Costa Rica**	Cahuita	NA	NA	*Pontoscolex corethrurus*	ND	?/1	Lapied and Lavelle (2003)
**Cuba**	Boyeros	Organic	Monoculture	*Dichogaster affinis*, *Dichogaster bolaui*, *Onychochaeta elegans*, *Polypheretima elongata*, *Protozapotecia angelesae*	5	2/3	Martínez-Leiva (2002)
**Guadeloupe (France)**	Capesterre-Belle-Eau	NA	Monoculture	*Pontoscolex corethrurus*	ND	?/1	Lafont et al. (2007)
	Capesterre-Belle-Eau (Gloria Bas)	Conventional	Monoculture	*Pontoscolex corethrurus*	1	0/1	Burac et al. (2018)
	Capesterre-Belle-Eau (Source)	Conventional	Monoculture	*Pontoscolex corethrurus*	1	0/1	Burac et al. (2018)
	Capesterre-Belle-Eau (Bergerie)	Agroecological	Monoculture	*Pontoscolex corethrurus*, unknown sp. 2	2	?	Burac et al. (2018)
	Baillilf (Sextius)	Agroecological	Monoculture	*Pontoscolex corethrurus*, unknown sp.	2	?	Burac et al. (2018)
	Baillilf (Grand Canon)	Agroecological	Monoculture	*Pontoscolex corethrurus*, unknown sp. 3	2	?	Burac et al. (2018)
	Saint-Claude (Saut d’Eau)	Agroecological	Monoculture	*Pontoscolex corethrurus*	1	0/1	Burac et al. (2018)
**India**	Dakshina Kannada District (Belthangady)	NA	NA	*Hoplochaetella kempi*	ND	1/0	Siddaraju et al. (2013)
	Dakshina Kannada District (Mangalore)	NA	NA	*Konkadrilus bahli*	ND	1/0	Siddaraju et al. (2013)
	Dakshina Kannada District (Mangalore)	NA	NA	*Dichogaster affinis*	ND	0/1	Siddaraju et al. (2013)
**India**	Dakshina Kannada District (Bantwal)	NA	NA	*Octochaetona parva*	ND	1/0	Siddaraju et al. (2010)
	Dakshina Kannada District (sites not detailed)	NA	NA	*Amynthas corticis*, *Hoplochaetella kempi*, *Hoplochaetella stuarti*, *Hoplochaetella suctoria*, *Megascolex konkanensis*, *Metaphire posthuma*, *Octochaetona paliensis*, *Octochaetona parva*	ND	7/1	Siddaraju et al. (2010, 2013)
	Kerala (Vellayambalam)	NA	NA	*Perionyx excavatus*, *Pontoscolex corethrurus*	2	0/2	Nair et al. (2007)
	Mizoram	NA	Monoculture	*Drawida nepalensis*, *Drawida rangamatiana*, *Drawida* sp., *Metaphire houlleti*, *Perionyx excavatus*	5	3/2	Lalthanzara (2007)
	Mizoram	NA	Polyculture	*Drawida nagana*, *Drawida* sp., *Metaphire houlleti*, *Perionyx excavatus*	4	2/2	Lalthanzara (2007)
	Rajapalayam	NA	NA	*Lampito mauritii*, *Perionyx excavatus*	2	1/1	Mariappan et al. (2013)
	Udupi District (Adve)	NA	NA	*Megascolex konkanensis*	1	1/0	Kumar et al. (2018)
	Udupi District (Adve)	NA	NA	*Metaphire houlleti*	1	0/1	Kumar et al. (2018)
	Udupi District (Bellibetu)	NA	NA	*Metaphire houlleti*, *Pontoscolex corethrurus*	2	0/2	Kumar et al. (2018)
	Udupi District (Mudarangadi)	NA	NA	*Pontoscolex corethrurus*	1	0/1	Kumar et al. (2018)
	Udupi District (Nandikur)	NA	NA	*Drawida ampullacea*, *Drawida sulcata*, *Metaphire peguana*	3	3/0	Kumar et al. (2018)
	Udupi District (Nandikur)	NA	NA	*Drawida ampullacea*	1	1/0	Kumar et al. (2018)
	Udupi District (Padabettu)	NA	NA	*Perionyx excavatus*	1	0/1	Kumar et al. (2018)
	Udupi District (Yellur)	NA	NA	*Mallehulla indica*, *Megascolex konkanensis*	2	2/0	Kumar et al. (2018)
	West Tripura (Mohanpur, Maheshkhola, Rastermatha)	Organic	Monoculture	*Amynthas alexandri*, *Drawida assamensis*, *Drawida papillifer*, *Eutyphoeus comillahnus*, *Lampito mauritii*, *Lennogaster* sp., *Metaphire houlleti*, *Metaphire posthuma*, *Octochaetona beatrix*, *Perionyx excavatus*, *Pontoscolex corethrurus*	3–7	4/7	Dhar and Chaudhuri (2018)
**Indonesia**	Bangkalan (Kamal, Burneh, Socah, Bypass)	NA	NA	*Amynthas robustus*, *Metaphire californica*, *Metaphire javanica*	ND	1/2	Budijastuti (2019)
	Bangkalan (Tanah Merah)	NA	NA	*Metaphire posthuma*	1	0/1	Budijastuti (2019)
	Bangkalan (Labang)	NA	NA	*Amynthas robustus*, *Metaphire javanica*, *Metaphire californica*, *Pheretima racemosa*	4	2/2	Budijastuti (2019)
	Gresik (Driyorejo, Kedamean, Ngipik, SumengkoLegundi)	NA	NA	*Amynthas robustus*, *Metaphire javanica*	ND	1/1	Budijastuti (2019)
	Gresik (Wringinanamon)	NA	NA	*Amynthas robustus*, *Metaphire javanica*, *Metaphire posthuma*	3	1/2	Budijastuti (2019)
	Sidoarjo (Waru, Taman, Sidoarjo, Tulangan, Tanggulangin, Candi)	NA	NA	*Amynthas robustus*, *Metaphire javanica*, *Metaphire posthuma*	ND	1/2	Budijastuti (2019)
	Surubaya (Pakal, Benowo, Tandes, Sukolilo, Gubeng, Gununganyar)	NA	NA	*Amynthas robustus*, *Metaphire javanica*, *Metaphire posthuma*	ND	1/2	Budijastuti (2019)
**Ivory Coast**	Lamto region	NA	NA	*Dichogaster wenkei*, *Reginaldia anomala*, *Stuhlmannia palustris*, *Stuhlmannia zielae*	ND	4/0	Tondoh (1994)
**Jamaica**	Clarendon, Crofts Mountain	NA	NA	*Drawida barwelli*, *Polypheretima elongata*	2	0/2	Sims (1987)
**Madagascar**	Ambatosoratra Ambatondrazaka	NA	NA	*Kynotus sihanakus*, *Kynotus* sp.2	2	2/0	Razafindrakoto et al. (2016), Csuzdi et al. (2017)
**Malaysia**	Serdang, Sengalor (Universiti Putra Malaysia)	NA	NA	*Pontoscolex corethrurus*	ND	0/1	Teng et al. (2006)
**Martinique (France)**	Le Lorrain (Feugère)	Conventional	Monoculture	*Pontoscolex corethrurus*	1	0/1	Burac et al. (2018)
	Le Lorrain (Limite)	Agroecological	Monoculture	*Pontoscolex corethrurus*	1	0/1	Burac et al. (2018)
	Le Lorrain (Bellevue)	Conventional	Monoculture	*Pontoscolex corethrurus*	1	0/1	Burac et al. (2018)
	L’Ajoupa-Bouillon (Allée Domergue 3)	Agroecological	Monoculture	*Pontoscolex corethrurus*	1	0/1	Burac et al. (2018)
	Basse-Pointe (Fromager Rivière)	Conventional	Monoculture	*Pontoscolex corethrurus*	1	0/1	Burac et al. (2018)
	Basse-Pointe (Dantu Bas)	Agroecological	Monoculture	*Pontoscolex corethrurus*	1	0/1	Burac et al. (2018)
**Mexico**	Tabasco	NA	Monoculture	*Lavellodrilus bonampakensis*, *Pontoscolex corethrurus*	2	1/1	Huerta et al. (2005)
	Tabasco, Teapa B1	NA	Monoculture	*Balanteodrilus pearsei*, *Drawida barwelli*, *Polypheretima elongata*, *Pontoscolex corethrurus*, *Pontoscolex* sp.	5	1/4	Geissen et al. (2009)
	Tabasco, Teapa B2	NA	Monoculture	*Balanteodrilus pearsei*, *Dichogaster bolaui*, *Drawida barwelli*, *Periscolex brachycystis*, *Polypheretima elongata*, *Pontoscolex* sp.	6	2/4	Geissen et al. (2009)
	Tabasco, Teapa AF1	Agroforestry	Polyculture	*Balanteodrilus pearsei*, *Dichogaster bolaui*, *Drawida barwelli*, *Polypheretima elongata*, *Pontoscolex corethrurus*, *Pontoscolex* sp.	6	2/4	Geissen et al. (2009)
	Tabasco, Teapa AF2	Agroforestry	Polyculture	*Balanteodrilus pearsei*, *Dichogaster bolaui*, *Drawida barwelli*, *Polypheretima elongata*, *Pontoscolex corethrurus*	5	1/4	Geissen et al. (2009)
	Tabasco, Teapa (site 1)	Conventional	NA	*Dichogaster saliens*, *Diplotrema murchiei*, *Pontoscolex corethrurus*	3	½	Huerta et al. (2013)
	Tabasco, Teapa (site 2)	Conventional	NA	*Dichogaster saliens*, *Pontoscolex corethrurus*	2	0/2	Huerta et al. (2013)
	Tabasco, Teapa (site 3)	Conventional	NA	*Diplotrema murchiei*, *Polypheretima elongata*, *Pontoscolex corethrurus*	3	1/2	Huerta et al. (2013)
	Tabasco, Teapa (site 4)	Conventional	Polyculture	*Amynthas gracilis*, *Pontoscolex corethrurus*	2	0/2	Huerta et al. (2007)
	Tabasco, Teapa (site 5)	Conventional	Polyculture	*Dichogaster saliens*, *Polypheretima elongata*, *Pontoscolex corethrurus*	3	0/3	Huerta et al. (2013)
	Tabasco, Teapa (site 6)	Conventional	NA	*Dichogaster saliens*, *Pontoscolex corethrurus*	2	0/2	Huerta et al. (2007)
	Tabasco, Pablo L. Sidar	NA	Monoculture	*Lavellodrilus bonampakensis*, *Pontoscolex corethrurus*	2	1/1	Huerta et al. (2013)
	Tamaulipas (Biosphere Reserve “El Cielo”)	NA	NA	*Amynthas gracilis*	ND	0/1	Barois (1992)
	Actopan, Ejido Buenavista	NA	NA	*Balanteodrilus psammophilus*	ND	1/0	Fragoso and Rojas (2007)
**Nicaragua**	Managua	NA	NA	*Dichogaster bolaui*, *Periscolex brachycystis*	2	1/1	Sherlock et al. (2011)
**Peru**	Sarita Colonia	NA	Monoculture	*Pontoscolex corethrurus* and two native spp.	3	2/1	Pashanasi (2007)
**Philippines**	Davao (Sumitomo Fruits Corporation, 15% site)	Conventional	Monoculture	*Metaphire* sp., *Pithemera bicincta*, *Pontoscolex corethrurus*	3	1/2	Fusilero et al. (2013)
	Davao (Sumitomo Fruits Corporation, 25% site)	Conventional	Monoculture	*Metaphire cai*, *Metapheretima* sp., *Perionyx excavatus*	3	2/1	Fusilero et al. (2013)
**Portugal**	Madeira Island (Ribeira Brava)	NA	NA	*Aporrectodea moebii*, *Eisenia eisensi*, *Metaphire californica*	3	0/3	Talavera (1996)
	Madeira Island (Funchal)	NA	NA	*Amynthas gracilis*, *Metaphire californica*, *Ocnerodrilus occidentalis*	3	0/3	Talavera (1996)
**Portugal**	Madeira Island (Santa Cruz)	NA	NA	*Amynthas gracilis*	1	0/1	Talavera (1996)
	Madeira Island (Terceira Lombada)	NA	NA	*Aporrectodea moebii*, *Eiseniella tetraedra*	2	0/2	Talavera (1996)
	Madeira Island (Porto Moniz)	NA	NA	*Amynthas gracilis*, *Aporrectodea rosea*, *Aporrectodea trapezoides*, *Dendrobaena pseudohortensis*	4	0/4	Talavera (2011)
	Madeira Island (Terceira Lombada)	NA	NA	*Aporrectodea caliginosa*, *Aporrectodea rosea*, *Eiseniella tetraedra*	3	0/3	Talavera (2011)
**Seychelles**	Cousine Island	NA	Monoculture	*Pontoscolex corethrurus*	ND	0/1	Plisko (2001)
**South Africa**	KwaZulu-Natal (Fairfield Farm)	NA	Monoculture	*Pontoscolex corethrurus*	ND	0/1	Plisko (2001)
	KwaZulu-Natal (Benhurst Farm)	NA	Monoculture	*Pontoscolex corethrurus*	ND	0/1	Plisko (2001)
	KwaZulu-Natal (6 sites in Eshowe)	NA	Monoculture	*Amynthas corticis*, *Amynthas minimus*, *Amynthas rodericensis*, *Dichogaster bolaui*, *Pontoscolex corethrurus*, and one other sp.	ND	0/5	Dlamini and Haynes (2004)
**Spain**	Gomera Island (Agulo)	NA	NA	*Amynthas rodericensis*, *Allolobophora chlorotica*, *Eiseniella tetraedra*, *Ocnerodrilus occidentalis*	4	0/4	Talavera (1990a, 2007)
	Gomera Island (Barranco de la Villa)	NA	NA	*Bimastos rubidus*, *Ocnerodrilus occidentalis*, *Pithemera bicincta*	3	0/3	Talavera (2007)
	Gomera Island (Barranco del Valle)	NA	NA	*Allolobophora chlorotica*, *Metaphire californica*	2	0/2	Talavera (1990b, 2007)
	Gomera Island (Casas de Aluce)	NA	NA	*Aporrectodea rosea*, *Microscolex phosphoreus*	2	0/2	Talavera (2007)
	Gomera Island (Cabo Verde)	NA	NA	*Amynthas gracilis*, *Bimastos rubidus*	2	0/2	Talavera (1990b, 2007)
	Gomera Island (Costa Agulo)	NA	NA	*Aporrectodea trapezoides*, *Amynthas rodericensis*, *Bimastos rubidus*, *Ocnerodrilus occidentalis*	4	0/4	Talavera (2007)
	Gomera Island (El Molinito)	NA	NA	*Amynthas morrisi*, *Microscolex phosphoreus*	2	0/2	Talavera (2007)
	Gomera Island (Hermigua)	NA	NA	*Aporrectodea rosea*, *Bimastos rubidus*, *Eisenia fetida*, *Ocnerodrilus occidentalis*	4	0/4	Talavera (1990a, 2007)
	Gomera Island (Laguna de Santiago)	NA	NA	*Amynthas morrisi*, *Aporrectodea rosea*, *Aporrectodea trapezoides*, *Bimastos rubidus*, *Dendrobaena hortensis*, *Dichogaster affinis*, *Metaphire californica*, *Pithemera bicincta*	9	0/9	Talavera (2007)
	Gomera Island (Playa de Santiago)	NA	NA	*Ocnerodrilus occidentalis*	1	0/1	Talavera (1990a)
	Gomera Island (Seimal)	NA	NA	*Eiseniella tetraedra*, *Metaphire californica*, *Microscolex phosphoreus*	3	0/3	Talavera (2007)
	Gomera Island (Taguluche)	NA	NA	*Amynthas morrisi*, *Allolobophora chlorotica*, *Octalasion lacteum*	3	0/3	Talavera (2007)
	Gomera Island (Valle Gran Rey)	NA	NA	*Allolobophora chlorotica*, *Aporrectodea trapezoides*, *Dendrobaena hortensis*, *Eisenia fetida*, *Microscolex dubius*, *Pithemera bicincta*	5	0/5	Talavera (2007)
	Gran Canaria (Lomo del Galeón)	NA	NA	*Ocnerodrilus occidentalis*	1	0/1	Talavera (1990a)
	Gran Canaria (Los Llanos)	NA	NA	*Ocnerodrilus occidentalis*, *Pithemera bicincta*	2	0/2	Talavera (1990a)
	Gran Canaria Island (Bañaderos)	NA	NA	*Metaphire californica*	1	0/1	Talavera (1990b)
	Gran Canaria Island (Barranco Guiniguada)	NA	NA	*Amynthas morrisi*	1	0/1	Talavera (1990b)
	Gran Canaria Island (Frontón)	NA	NA	*Amynthas gracilis*	1	0/1	Talavera (1990b)
	Gran Canaria Island (Galdar)	NA	NA	*Amynthas morrisi*	1	0/1	Talavera (1990b)
**Spain**	Gran Canaria Island (Hoya Mondondo)	NA	NA	*Pithemera bicincta*	1	0/1	Talavera (1990b)
	Gran Canaria Island (La Aldea)	NA	NA	*Dichogaster affinis*	1	0/1	Talavera (1992b)
	Gran Canaria Island (Pedrazo)	NA	NA	*Pithemera bicincta*	1	0/1	Talavera (1990b)
	Gran Canaria Island (Tenoya)	NA	NA	*Amynthas morrisi*	1	0/1	Talavera (1990b)
	Hierro Island (Los Mocanes)	NA	NA	*Ocnerodrilus occidentalis*	1	0/1	Talavera (1990a)
	Hierra Island (NE tip)	NA	NA	*Microscolex phosphoreus*	ND	0/1	Talavera and Pérez (2009)
	La Palma Island (Barranco de las Angustias)	NA	NA	*Amynthas gracilis*	1	0/1	Talavera (1990b)
	La Palma Island (Barranco Nogales)	NA	NA	*Amynthas gracilis*	1	0/1	Talavera (1990b)
	La Palma Island (El Socorro)	NA	NA	*Pithemera bicincta*	1	0/1	Talavera (1990b)
	La Palma Island (La Caldereta)	NA	NA	*Amynthas morrisi*, *Metaphire californica*	2	0/2	Talavera (1990b)
	La Palma Island (Los Cancajos)	NA	NA	*Amynthas morrisi*	1	0/1	Talavera (1990b)
	La Palma Island (Los Llanos de Aridane)	NA	NA	*Amynthas morrisi*, *Metaphire californica*	2	0/2	Talavera (1990b)
	La Palma Island (Tazacorte)	NA	NA	*Amynthas gracilis*, *Amynthas morrisi*, *Metaphire californica*	3	0/3	Talavera (1990b)
	Tenerife Island (Abama)	NA	Monoculture	*Aporrectodea rosea*, *Dendrobaena hortensis*, *Eisenia andrei*, *Microscolex dubius*	4	0/4	Talavera (1992a)
	Tenerife Island (Adeje)	NA	NA	*Ocnerodrilus occidentalis*	1	0/1	Talavera (1990a)
	Tenerife Island (Bajamar)	NA	Monoculture	*Amynthas morrisi*, *Aporrectodea rosea*, *Dichogaster affinis*, *Eisenia andrei*, *Microscolex phosphoreus*, *Ocnerodrilus occidentalis*	6	0/6	Talavera (1990a, 1992a, 1992b)
	Tenerife Island (Barranco de Santos)	NA	Monoculture	*Amynthas morrisi*, *Aporrectodea rosea*, *Bimastos rubidus*, *Eisenia andrei*, *Microscolex dubius*, *Pithemera bicincta*	6	0/6	Talavera (1990b, 1992a)
	Tenerife Island (Barranco del Inglés)	NA	Monoculture	*Aporrectodea rosea*, *Aporrectodea trapezoides*, *Eisenia andrei*, *Microscolex dubius*	4	0/4	Talavera (1992a)
	Tenerife Island (Barranco la Atalaya)	NA	Monoculture	*Aporrectodea rosea*, *Pithemera bicincta*	2	0/2	Talavera (1992a)
	Tenerife Island (Barranco las Galletas)	NA	Monoculture	*Aporrectodea rosea*, *Eisenia andrei*, *Ocnerodrilus occidentalis*	3	0/3	Talavera (1992a)
	Tenerife Island (Barranco San Felipe)	NA	Monoculture	*Amynthas gracilis*, *Eisenia andrei*, *Pithemera bicincta*	3	0/3	Talavera (1992a, 1990b)
	Tenerife Island (Buenavista del Norte)	NA	NA	*Ocnerodrilus occidentalis*	1	0/1	Talavera (1990a)
	Tenerife Island (Casablanca)	NA	Monoculture	*Amynthas corticis*, *Aporrectodea rosea*, *Eisenia andrei*, *Ocnerodrilus occidentalis*	4	0/4	Talavera (1992a)
	Tenerife Island (Costa Valle Guerra)	NA	Monoculture	*Amynthas gracilis*	1	0/1	Talavera (1992a)
	Tenerife Island (El Puente)	NA	Monoculture	*Amynthas gracilis*, *Aporrectodea rosea*, *Eisenia andrei*, *Microscolex phosphoreus*, *Ocnerodrilus occidentalis*	5	0/5	Talavera (1992a)
	Tenerife Island (El Rincón)	NA	Monoculture	*Amynthas gracilis*, *Bimastos rubidus*, *Dendrobaena cognetti*, *Microscolex dubius*, *Microscolex phosphoreus*, *Octodrilus complanatus*	6	0/6	Talavera (1992a)
**Spain**	Tenerife Island (El Socorro)	NA	NA	*Pithemera bicincta*	1	0/1	Talavera (1990b)
	Tenerife Island (Fañabé)	NA	Monoculture	*Amynthas corticis*, *Aporrectodea rosea*, *Dichogaster affinis*, *Eisenia andrei*, *Ocnerodrilus occidentalis*	5	0/5	Talavera (1990a, 1992a, 1992b)
	Tenerife Island (Güimar)	NA	NA	*Dichogaster affinis*, *Ocnerodrilus occidentalis*	2	0/2	Talavera (1990a, 1992b)
	Tenerife Island (Iboybo)	NA	Monoculture	*Aporrectodea rosea*, *Eisenia andrei*, *Ocnerodrilus occidentalis*	3	0/3	Talavera (1992a)
	Tenerife Island (Icod de Los Vinos)	NA	Monoculture	*Dendrobaena cognetti*, *Bimastos rubidus*, *Octodrilus complanatus*, *Ocnerodrilus occidentalis*	4	0/4	Talavera (1992a)
	Tenerife Island (Igueste)	NA	Monoculture	*Allolobophora chlorotica*, *Aporrectodea rosea*, *Aporrectodea trapezoides*, *Pontoscolex corethrurus*, *Ocnerodrilus occidentalis*	5	0/5	Talavera (1992a)
	Tenerife Island (La Hondura)	NA	Monoculture	*Amynthas morrisi*	1	0/1	Talavera (1992a)
	Tenerife Island (La Longuera)	NA	Monoculture	*Amynthas morrisi*, *Aporrectodea rosea*, *Eisenia fetida*, *Microscolex dubius*, *Octodrilus complanatus*	5	0/5	Talavera (1992a)
	Tenerife Island (La Matanza)	NA	Monoculture	*Bimastos rubidus*, *Eisenia andrei*, *Microscolex phosphoreus*	3	0/3	Talavera (1992a)
	Tenerife Island (La Montañeta)	NA	NA	*Pithemera bicincta*	1	0/1	Talavera (1990b)
	Tenerife Island (La Vera)	NA	Monoculture	*Bimastos rubidus*, *Eisenia andrei*, *Microscolex phosphoreus*	3	0/3	Talavera (1992a)
	Tenerife Island (Las Arenas)	NA	NA	*Amynthas morrisi*	1	0/1	Talavera (1990b)
	Tenerife Island (Las Galletas)	NA	Monoculture	*Eisenia andrei*, *Bimastos eiseni*, *Ocnerodrilus occidentalis*	3	0/3	Talavera (1990a, 1992a)
	Tenerife Island (Las Madrigueras)	NA	NA	*Amynthas morrisi*	1	0/1	Talavera (1990b)
	Tenerife Island (Los Quintos)	NA	Monoculture	*Dendrobaena cognetti*, *Bimastos rubidus*, *Microscolex phosphoreus*, *Pithemera bicincta*, *Ocnerodrilus occidentalis*	5	0/5	Talavera (1992a)
	Tenerife Island (Los Realejos)	NA	NA	*Pithemera bicincta*	1	0/1	Talavera (1990b)
	Tenerife Island (Los Rechazos)	NA	Monoculture	*Aporrectodea trapezoides*, *Bimastos rubidus*, *Eisenia fetida*, *Octodrilus complanatus*, *Pithemera bicincta*	5	0/5	Talavera (1992a)
	Tenerife Island (Los Silos)	NA	Monoculture	*Amynthas morrisi*, *Aporrectodea rosea*, *Dichogaster affinis*, *Eisenia andrei*, *Ocnerodrilus occidentalis*	5	0/5	[Bibr B108][Bibr B109])
	Tenerife Island (Loss Llanos)	NA	Monoculture	*Amynthas morrisi*, *Bimastos rubidus*, *Eisenia andrei*, *Pithemera bicincta*	4	0/4	Talavera (1992a)
	Tenerife Island (Playa de las Aguas)	NA	Monoculture	*Amynthas morrisi*, *Eisenia andrei*, *Pithemera bicincta*	3	0/3	Talavera (1992a)
	Tenerife Island (Playa de San Juan)	NA	Monoculture	*Aporrectodea rosea*, *Dendrobaena hortensis*, *Bimastos rubidus*, *Eisenia andrei*	4	0/4	Talavera (1992a)
	Tenerife Island (Playa San Marcos)	NA	Monoculture	*Pithemera bicincta*, *Bimastos rubidus*, *Microscolex phosphoreus*	3	0/3	Talavera (1992a)
	Tenerife Island (Puertito de Gilimar)	NA	Monoculture	*Microscolex phosphoreus*, *Pithemera bicincta*, *Ocnerodrilus occidentalis*	3	0/3	Talavera (1992a)
	Tenerife Island (Puerto de Santiago)	NA	Monoculture	*Amynthas morrisi*	1	0/1	Talavera (1990b, 1992a)
	Tenerife Island (Punta del Hidalgo)	NA	Monoculture	*Amynthas gracilis*, *Ocnerodrilus occidentalis*	2	0/2	Talavera (1992a)
	Tenerife Island (San Andrés)	NA	Monoculture	*Amynthas morrisi*, *Aporrectodea rosea*, *Microscolex phosphoreus*, *Ocnerodrilus occidentalis*	4	0/4	Talavera (1992a)
	Tenerife Island (San Bernardo)	NA	Monoculture	*Amynthas corticis*, *Amynthas morrisi*, *Aporrectodea rosea*, *Eisenia andrei*	4	0/4	Talavera (1992a)
**Spain**	Tenerife Island (San Juan de la Rambla)	NA	Monoculture	*Amynthas gracilis*, *Bimastos rubidus*, *Dendrobaena hortensis*, *Eisenia fetida*, *Pithemera bicincta*	5	0/5	Talavera (1990b, 1992a)
	Tenerife Island (San Pedro de Daute)	NA	Monoculture	*Amynthas morrisi*, *Aporrectodea rosea*, *Ocnerodrilus occidentalis*	3	0/3	Talavera (1992a)
	Tenerife Island (Santo Domingo)	NA	Monoculture	*Dendrobaena cognetti*, *Microscolex dubius*, *Microscolex phosphoreus*	3	0/3	Talavera (1992a)
	Tenerife Island (Taganana)	NA	Monoculture	*Amynthas morrisi*	1	0/1	Talavera (1992a)
	Tenerife Island (Tejina)	NA	Monoculture	*Amynthas corticis*, *Pithemera bicincta*	2	0/2	Talavera (1992a)
**Taiwan**	Central region	NA	NA	*Pontoscolex corethrurus*	ND	0/1	Tsai et al. (2000)
**Uganda**	Kabanyolo University Farm	NA	NA	*Dichogaster* sp. 1, *Dichogaster* sp. 2, *Gordiodrilus* sp., *Pygmaeodrilus* sp., *Polytoreutus* sp. 1	5	5/0	Block and Banage (1968)

Overall, ≥104 earthworm species from 10 earthworm families were recorded from banana/plantain fields worldwide, of which around 61 (59%) were native and 43 exotic to the sampling sites (Table 5). Estimating these numbers is difficult due to insufficient taxonomic resolution in some samples, as well as the uncertain origin of some widespread anthropochores (peregrines transported by humans), particularly in the Megascolecidae family (Blakemore 2002). Highest species richness (27) was observed overall in India, where most of the species found were native (74%). High proportions of native species were also observed in Ivory Coast, Madagascar, and Uganda (possibly 100%) as well as Cameroon (75%), but were lower in Mexico (58%) and Colombia (53%). In these countries, many of the plantations were managed more traditionally, or using agroforestry, although the low number of sampling sites may also be responsible for these high values, particularly in the former countries. In fact, agroforestry systems had a total of 22 species from nine sites, while conventional production systems had only nine species from 13 sites. Nonetheless, because not enough information was provided in the publications on management practices (not reported in ≥150 sites; Table 4), the role of less intensive banana production systems in maintaining native earthworm populations must still be further evaluated.

High species richness was also detected overall in Spain (25), mainly due to the higher sampling effort involving a large number of sites in the Canary Islands. However, all of the species encountered on the islands offshore of Africa were exotic, their introduction having been stimulated over centuries of human colonization bringing in exotic soils and crops (Talavera 2007, 2011). The Caribbean islands had few species (5), despite a large sampling effort, and many sites were dominated by *P. corethrurus* (Burac et al. 2018). In Brazil, Costa Rica, Martinique, Jamaica, Bermuda, the Seychelles, Taiwan, Malaysia, and China, all the earthworm species encountered were exotic (Table 5). The continent with the highest number of species recorded was Africa (50), of which 40% were native. In Asia, 35 species were recorded, with a higher proportion of natives (66%). In North and South America, around 50% of the species found were native, but these were mainly due to the higher number of natives observed in Colombian (Feijoo et al. 2018) and Mexican (Geissen et al. 2009; Huerta et al. 2013) plantations.

**Table 5.** Number of quantitative (with abundance data) and qualitative (where species were identified) sampling sites and earthworm species (total, native, and exotic) and families found in banana plantations in different countries of the world.

Country	No. sites: Quant./Qual.^1^	Total No. species	Native	Exotic	Families
**Asia**	**6/≥47**	**35**	**22**	**13**	**5**
Bangladesh	0/1	3	1	2	2
China	0/1	1	0	1	1
India	4/≥20	27	20	7	5
Indonesia	0/23	5	1	4	1
Malaysia	0/1	1	0	1	1
Philippines	2/2	6	3	3	2
Taiwan	0/1	1	0	1	1
**Africa**	**33/97**	**50**	**20**	**30**	**7**
Cameroon	10/2	12	9	3	3
Canary Islands (Spain)^2^					
*Gomera*	0/13	18	0	18	4
*Gran Canaria*	0/10	6	0	6	3
*Hierro*	0/2	2	0	2	2
*La Palma*	0/7	3	0	3	1
*Tenerife*	0/45	19	0	19	5
Ivory Coast	1/1	4	4	0	3
Madagascar	0/1	2	2	0	1
Madeira (Portugal)^2^	0/6	10	0	10	3
Seychelles	0/1	1	0	1	1
South Africa	6/8	5	0	5	3
Uganda	6/1	5	5	0	3
**North America**	**12/16**	**14**	**7**	**7**	**4**
Bermuda	0/2	2	0	2	1
Mexico	12/14	12	5	7	4
**Central America/Caribbean**	**53/≥17**	≥**10**	**4**	≥**6**	**4**
Costa Rica	≥5/1	1	0	1	1
Cuba	1/1	5	2	3	3
Dominica	1/0	2	1?	1	2
Guadeloupe (France)	40/7	4?	?	≥1	≥1
Martinique (France)	6/6	1	0	1	1
Jamaica	0/1	2	0	2	2
Nicaragua	0/1	2	1	1	2
**South America**	**49/33**	**20**	**10**	**10**	**6**
Brazil	16/16	7	0	7	5
Colombia	32/15	15	8	7	4
Peru	1/1	3	2	1	≥1
**Total**	**153/210**	≥**104**	≥**61**	≥**43**	**10**

^1^Quant.=quantitative samples, taken using various sampling methods (mostly hand sorting of soil monoliths); Qual.=qualitative samples, usually performed for biodiversity studies (species presence) and normally without specifying volume of soil sampled; ^2^Although politically these islands belong to Europe, biogeographically they belong to Africa.

Species richness in individual banana/plantain fields was measured in 166 of the 210 sites, and was generally very low, with an overall mean of 2.7 species per site worldwide, of which less than one (0.5) was native and 2.1 were exotic (full dataset in Cremonesi et al. 2020). Absolute richness in an individual plantation was highest in the banana plantations in Cameroon (Norgrove et al. 2011), where 8 and 10 species were found (Table 4), most of them native. The only other place with such high richness was a plantation in Gomera Island (Laguna de Santiago), where 9 species were found (Talavera 2007), although all of them were exotic. In West Tripura, up to 7 species were found in a banana plantation (Dhar and Chaudhuri 2018), but most plantations in the world had less than 3 species (~70% of sites), and the highest proportion was of sites with only 1 species (~30% of sites).

**Figure 3.**
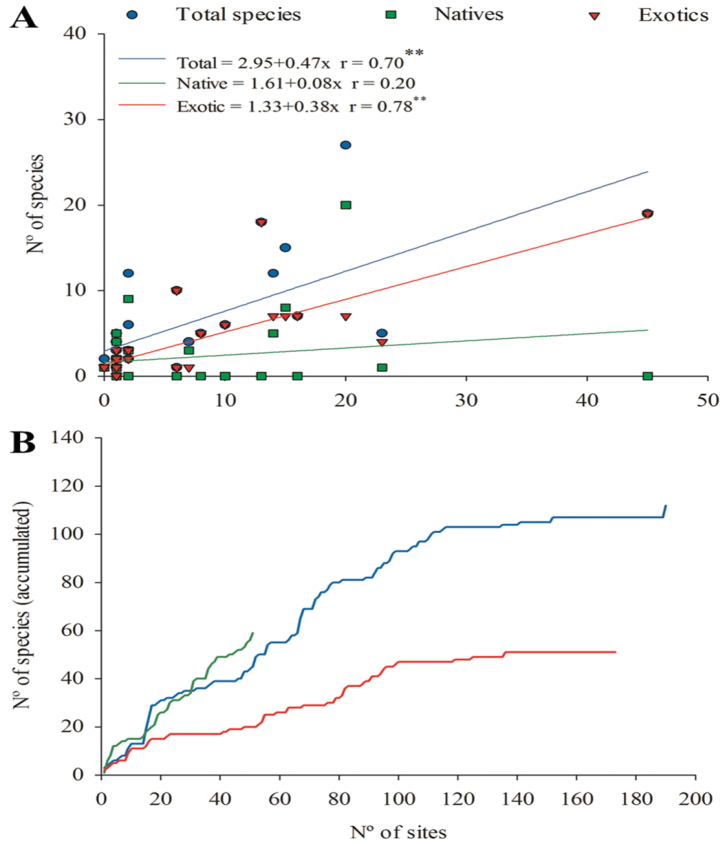
**A** Relationship between species richness (total, native, and exotic species) and the number of sampling sites in each world country (data from Table 5) and **B** Species accumulation curves for total, native and exotic species, depending on the number of sampling sites across the world. Linear regression equations and the value and significance (p value, with ** indicating p< 0.01) of the Pearson correlation coefficient (r) are provided in (**A**).

There was a clear positive relationship between the number of sites sampled in each country and the total number of species encountered (r = 0.7, p< 0.01), particularly for exotic (r = 0.78, p < 0.01) species (Fig. 3A). Although also positive, this relationship was not significant for native species. Nonetheless, the species accumulation curve for native species for all sampling sites in the world revealed a steep slope, that contrasts with the flattened-out accumulation curves for total and exotic species (Fig. 3B). This indicates that greater sampling efforts, particularly in more low-input production systems, especially in tropical countries with high earthworm biodiversity such as Ecuador (no studies with earthworms identified yet), Brazil and Colombia (Brown and James 2007; Feijoo 2007; Zicsi 2007) will certainly increase the number of species known from banana/plantain fields. Greater sampling efforts are also needed in other tropical countries with important plantain/banana production (FAO 2018), particularly when intercropped or in agroforestry systems (Norgrove et al. 2011; Norgrove and Hauser 2014), and where mostly native earthworm species may inhabit these fields, such as seen for Cameroon, Uganda and Ivory Coast. This phenomenon may likely also be applicable to other Western, Central and Eastern African countries, as well as many other Asian and Pacific countries, but the paucity of available data impedes further speculation.

Of the over 100 species found in banana and plantain fields worldwide, most belonged to the Megascolecidae (22%), Lumbricidae (17%) and Acanthodrilidae (16%) families (Cremonesi et al. 2020). These widespread exotic and often invasive species are found throughout the tropics and subtropics, and include several *Amynthas* and *Metaphire* spp. (Blakemore 2002). The most consistently recorded megascolecids were *A. gracilis* (6% of all records), *Amynthas morrisi* (Beddard, 1892) (5%), *Pithemera bicincta* (Perrier, 1875) (4%) and *Metaphire californica* (Kingerg, 1867), *Perionyx excavatus* Perrier, 1872 and *Polypheretima elongata* (Perrier, 1872) (all with 2% each) (Cremonesi et al. 2020). These megascolecids were found in over 15 countries, and were especially frequent in the Canary Islands. All of the lumbricids reported were exotic, and mainly found in the Canary and Madeira Islands (Spain, Portugal), with *Aporrectodea rosea* (Savigny, 1826) and *Eisenia andrei* Bouché, 1972 (both with ~4%) and *Bimastos rubidus* (Savigny, 1826) (3%) being the most frequently reported. Various octochaetid *Dichogaster* spp. of the Benhamiinae subfamily, i.e., Dichogaster (Diplothecodrilus) affinis (Michaelsen, 1890), D. (D.) bolaui (Michaelsen, 1891) and D. (D.) saliens (Beddard, 1893) (all with around 2% each) and the acanthodrilinae *Microscolex* spp., i.e., *Microscolex phosphoreus* (Dugés, 1837) with 3% and *M. dubius* (Fletcher, 1887) with 2% of records, were the most reported acanthodrilids. The *Dichogaster* spp. were found in 11 countries, mainly in Latin America and the Canary Islands, while the *Microscolex* spp. were found only in the Canary Island banana plantations. Similarly, the ocnerodrilid *Ocnerodrilus occidentalis* Eisen, 1878 with 6% of all records, was found in three countries (Brazil, Portugal, Spain), but most frequently in the Canary Islands.

The most commonly encountered earthworm species in banana plantations was *P. corethrurus* (11%), found in 15 countries, mainly in Latin America, but also in places as far away as South Africa, India, Bangladesh, Malaysia, Philippines and Taiwan. Interestingly, 37 out of 54 sites (69%) that identified earthworm species reported *P. corethrurus* as dominant in the banana plantations (Table 3).

Although *P. corethrurus* may affect soil physical properties negatively by increasing soil compaction under some conditions, it can also positively affect biogeochemical processes, microbial activity, plant production, and soil recovery (see review in Taheri et al. 2018). Furthermore, this species is known to reduce plant-parasitic nematode incidence in banana plants (Loranger-Merciris et al. 2012), and has also been known to promote beneficial plant growth-promoting bacteria in the rhizosphere (Braga et al. 2015). Hence, further work is warranted on the potential beneficial impacts of the presence and populations of *P. corethrurus* on banana plants, particularly considering its widespread distribution and high abundance in some locations (e.g., Costa Rica, Brazil, Guadeloupe, Martinique, Mexico). Several megascolecids such as *A. gracilis* are also known to affect soil physical and chemical properties in annual cropping systems (e.g., Peixoto and Marochi 1996; Bartz et al. 2010) as well as crop production (Brown et al. 1999), but little is known of their effects on banana plants. The latter statement is also valid for all of the other species most commonly found in banana plantations.

A total of 31 studies performed in 153 sites and 15 countries (Table 5) had quantitative earthworm data (on abundance and/or biomass) taken mainly by hand sorting soil monoliths of variable size (mostly 25 x 25 cm but sometimes larger, e.g., 50 × 50 cm) and occasionally using liquid extraction (e.g., formalin expulsion). Most of the study sites were in Guadeloupe (*N* = 40, of which 34 were by Clermont-Dauphin et al. (2004) and Colombia (*N* = 32; Molina and Feijoo 2017).

Overall earthworm abundance ranged from a minimum of 0 (Figueroa 2019) in an Ecuadorian plantation, to a maximum mean of over 1500 indiv. m^-2^ in banana plantations in Kwazulu-Natal, South Africa (Dlamini and Haynes 2004). Maximum biomass attained was 453.6 g m^-2^ for a site in West Tripura, India (Dhar and Chaudhuri 2018). Interestingly, a large number of sites (>50) had abundance values over 100 indiv. m^-2^, which could be considered quite high for earthworm density in annual agricultural crops (Bartz et al. 2013). Nonetheless, bananas are perennials often cultivated over several cropping cycles, allowing for reduced negative effects of soil preparation, and the soils are also often limed to correct pH and fertilized with inorganic fertilizers (mainly N, P and K) to promote soil fertility and banana production. In these conditions, earthworms present find a soil protected from rainfall impact, as well as frequent organic matter additions through the management of the banana trees, particularly where the residues are left on the soil surface. Consequently, their populations can increase rather rapidly over time, as observed by Okwakol (1994) in Uganda (Table 3).

These high earthworm abundances and biomasses may be contributing significantly to soil processes (bioturbation, nutrient cycling) in banana/plantain fields, as biomasses over 17 g m^-2^ and above 32 g m^-2^ are known to lead to moderate (20–40%) and important (>40%) grain production increases, respectively (Brown et al. 1999). Earthworm-induced improvement of plant health and production includes, e.g., plant-parasitic nematode population control (Lafont et al. 2007; Loranger-Merciris et al. 2012), high stable bioaggregate formation, creation of many galleries in the soil and enhanced nutrient mineralization (Lavelle 1997), all factors that deserve future attention. On the other hand, low earthworm abundance may be an indicator of soil degradation, or the use of inappropriate management practices, such as soil inversion or toxic pesticide use (Demetrio et al. 2019). This type of information could be used to help farmers with their management decisions, such as reduction in nematicide applications that reduce earthworm populations (Clermont-Dauphin et al. 2004).

Finally, 18 of the major banana-producing countries in the world (34 countries with >30,000 ha in production, or >1 Million T bananas produced yr^-1^; FAO 2018) were not examined in the present review due to lack of data. Hence, further sampling efforts are needed in order to provide adequate information on earthworm abundance and biodiversity in banana plantations in these countries, and to complement those reported here but with low sample intensity, particularly focusing on the presence of native species and/or large earthworm abundances, and to identify the reasons for these phenomena and their consequences for banana production and biodiversity conservation.

## Conclusions

Earthworms are an important component of banana and plantain fields worldwide and deserve further attention by taxonomists, ecologists and agronomists. Under some conditions, especially in lower-input polycultures, their abundance and biomass may reach high values and contribute significantly to soil processes and plant production. More than 70 studies performed in over 200 banana plantations of 28 countries found >100 species (around 60% of them native) from 10 families, although species richness in each sited tended to be low (generally <3 species) and exotic species predominated (particularly *P. corethrurus*). However, as many important banana-producing countries have not yet been evaluated, further work is warranted in order to better understand the earthworm communities and their functional roles in plantain/banana fields, and the role of management practices in affecting their populations and diversity worldwide.
